# Postactivation Potentiation Following Acute Bouts of Plyometric versus Heavy-Resistance Exercise in Collegiate Soccer Players

**DOI:** 10.1155/2018/3719039

**Published:** 2018-02-07

**Authors:** Sourabh Kumar Sharma, Shahid Raza, Jamal Ali Moiz, Shalini Verma, Irshad Husain Naqvi, Shahnawaz Anwer, Ahmad H. Alghadir

**Affiliations:** ^1^Centre for Physiotherapy and Rehabilitation Sciences, Jamia Millia Islamia, New Delhi, India; ^2^Dr. M. A. Ansari Health Centre, Jamia Millia Islamia, New Delhi, India; ^3^Rehabilitation Research Chair, College of Applied Medical Sciences, King Saud University, Riyadh, Saudi Arabia

## Abstract

Postactivation potentiation is referred to as an acute and temporary enhancement of muscle performance resulting from previous muscle contraction. The purpose of this study was to compare the acute effect of plyometric exercise (PLY) and heavy-resistance exercise (RES) on the blood lactate level (BLa) and physical performance. Fourteen male collegiate soccer players were randomized to perform either RES or PLY first and then crossed over to perform the opposite intervention. PLY consisted of 40 jumps, whereas RES comprised ten single repetitions at 90% of one repetition maximum. BLa and physical performance (countermovement jump height and 20-m sprint) were measured before and at 1 and 10 min following the exercise. No significant difference was observed in the BLa for both exercises (PLY and RES). Relative to baseline, countermovement jump (CMJ) height was significantly better for the PLY group after 1 min (*P* = 0.004) and after 10 min (*P* = 0.001) compared to that of the RES group. The 20-m sprint time was significantly better for PLY at 10 min (*P* = 0.003) compared to that of RES. The present study concluded that, compared to RES, PLY causes greater potentiation, which leads to improved physical performance. This trial is registered with NCT03150277.

## 1. Introduction

Postactivation potentiation (PAP) is the phenomenon by which muscular performance is acutely and temporarily enhanced because of muscular contractile history [1]. There is a considerable amount of literature recommending the application of a conditioning activity (CA) to augment the performance of a subsequent sprint, jump, and throw [1, 2]. Several studies have suggested that there are two fundamental mechanisms responsible for PAP: phosphorylation of myosin regulatory light chains (RLC) [1, 3, 4] and increased recruitment of higher-order motor units [1, 5]. Additionally, there is considerable evidence suggesting that changes in the pennation angle can influence PAP [2, 6]. As strength and conditioning professionals seek for new ways to increase the potential of their athletes, specifically muscular power, PAP may prove to be a useful tool. Previous literature is focused on what combinations of factors are most effective in eliciting PAP. These factors include modes of exercise used as a preconditioning activity, the rest interval between activity and performance, and the subject characteristics. While there is extensive literature on the factors involving PAP, there is no consensus on the ideal combination of these factors to optimize performance.

The most commonly used training techniques in sports are back squats and have been extensively used for eliciting a PAP response in either untrained, recreationally trained, or highly trained subjects [1, 2]. A lot of previous researches have made use of back-squat exercises with varying squat depths as a CA. Furthermore, research has also employed different types of load in CA with either repetition maximum (RM) loads [7] or submaximal loads performed at various percentages of 1 RM [8]. Similarly, plyometric exercise (PLY) has been used by top athletes for augmenting their jumping performance as well as enhancing muscular activation [9]. PLY are associated with the preferential recruitment of type II motor units [10], which is one central level mechanism underpinning PAP. Furthermore, PLY is capable of inducing levels of potentiation comparable to those induced through heavy-resistance exercise (RES) [11].

A detailed investigation of literature reveals that the muscle performance characteristics after an exercise depend upon the balance between fatigue and potentiation that exist together at variable degrees after completion of an exercise. When potentiation is dominating and fatigue is reduced, the muscle performance may improve. However, it does not change if fatigue and potentiation are at an equal level but decreases if fatigue is higher than potentiation [12]. Furthermore, literature supports the fact that plyometric CA may generate lower levels of fatigue compared to those generated by the loaded traditional-resistance exercise and, thus, result in higher potentiation while decreasing time required for achieving the maximal effect of PAP [13]. While previous researchers failed to report any significant difference between plyometric and heavy-resistance CA [14], a recent study revealed a greater effect of PAP on countermovement jump (CMJ) after heavy-resistance CA [11]. This discrepancy could be explained by the low volume of PLY employed that might be inadequate to induce high levels of potentiation. In light of these equivocal findings, the effect of contraction type on the fatigue and PAP process warrants further research while regulating recovery period following a CA and its volume.

Moreover, potentiation can be realized at some point during the period of recovery as fatigue subsides at a faster rate than PAP. The various mechanisms proposed for fatigue elicited after an RES are depletion of substrate [15], buildup of hydrogen ions [16], and mechanical disruption of the myofibrillar architecture [17]. However, there is a dearth of investigations examining the relation between potentiation and fatigue with a physiological marker. As exercise induces interstitial acidification, which has been postulated to be the limiting factor for physical performance at short durations [18], and there exists an inverse linear relationship between blood lactate level (BLa) and blood pH [19], it seems rational to use BLa as an indicator for exercise-induced fatigue. Therefore, the current research aimed to compare the acute effects of PLY and RES on PAP in soccer players and simultaneously investigate the PAP–fatigue relationship.

## 2. Materials and Methods

### 2.1. Subjects

Fourteen male collegiate soccer players (age = 18.57 ± 0.94 years, height = 172.21 ± 5.07 cm, and mass = 64.79 ± 7.98 kg) volunteered to participate in the study and an informed consent was obtained. The number of subjects was determined using the G^*∗*^Power software (version 3.1.9.2; Heinrich-Heine-Universität Düsseldorf, Germany) from the changes in sprint performance in the systematic review on PAP done by Seitz and Haff [13], and 14 subjects (considering the 12% dropout) were shown to be necessary based on the effect size = 0.51, *α* = 0.05, and power (1 − *β*) = 0.95. Inclusion criteria were at least 2 years of playing experience in soccer, sport-specific training on at least 2 occasions per week and playing competitively once per week, and no previous experience of PLY or RES. Exclusion criteria were lower-extremity reconstructive surgery in the last 24 months or unresolved musculoskeletal disorder, which would prohibit players to participate in the sport, and consumption (or prior consumption) of growth hormone, anabolic steroids, or any kind of performance-enhancing drugs. Ethical clearance was taken from the Institutional Ethical Committee of Jamia Millia Islamia.

### 2.2. Testing Procedures

Each subject had to report for assessment after a minimum of one day of rest from training or competition of all sorts. All tests were done between 09:00 and 11:00 AM to control any diurnal variation. Also, all subjects were instructed to avoid caffeine intake on the testing day and one day before testing. The procedure was performed in three separate sessions. In the first session, subjects were familiarized with the experimental conditions and 1 RM was calculated. Subjects were then randomized to perform either RES or PLY first and then crossed over to perform the other intervention after a washout period of 48 hours. To determine the order effect, one group (*n* = 7) performed RES first and then PLY, whereas the other group (*n* = 7) performed PLY first and then RES.

The procedure began with warm-up. The warm-up protocol consisted of 5 min submaximal running at 9 km h^−1^ followed by 5 min of light stretching of the lower limb and half squat with low loads (two sets of five repetitions at 50% of body mass with 2 min of rest between sets) [11], and after 1 minute of recovery, baseline measures of CMJ height, 20-m sprint time, and BLa were recorded. The subjects then performed RES or PLY 3 min after baseline measurement. Subsequently, CMJ height, 20-m sprint time, and BLa were recorded 1 min and 10 min after the exercise. The testing days were interspersed with a minimum of 48 h rest period to limit the effects of fatigue on subsequent tests.

### 2.3. Protocol

PLY consisted of two sets of ten ankle hops, three sets of five hurdle hops, and five drop jumps from 50 cm height. Ankle hops are the bilateral exercises performed with stiff leg action and a rapid reflexive bounce back off the ground. Hurdle hops involve tuck-jump action to go over a hurdle of 70 cm while aiming at spending a very small amount of time on the floor between each jump. In drop jumps, the subject drops down from a box onto the mat, bending the knees on landing and then immediately going for a maximal vertical jump. Thirty-second rest was given between the sets of ankle hops and hurdle hops with 15 s rest between repetitions of the drop jump [20].

RES consisted of ten single repetitions of back half squats at 90% of 1 RM. In 1-RM estimation, before testing, subjects performed warm-up consisting of two sets of ten and eight repetitions at loads of 50–80% of self-identified 1 RM. The estimation of original 1 RM started from a value of 5% less than the one declared by the subjects, and the load was increased (2%) after successful trial. This procedure was continued until the subject failed to lift the given load and demonstrated inability to perform a full range of motion. The rest period between the sets was 3 min, and approximately 3–6 trials were executed for the final prediction of 1 RM [21]. Nevertheless, all the methods for the tests were examined and supervised in accordance with the American College of Sports Medicine (ACSM) guidelines. Each subject maintained an upright standing posture, looked ahead, and firmly held the bar with both hands. This bar was supported on the shoulders, and the subject later bent his knees until 90°, followed by raising himself to the standing posture with the extension of the lower limbs [22]. All participant successfully completed 10 repetitions owing to a better aerobic profile by virtue of soccer training that enabled them to tolerate a high number of repetitions before fatigue [23, 24].

### 2.4. Criterion Measures

For BLa, a small blood sample was taken from the fingertip before exercise and 1 and 10 min after the exercise, and BLa was measured by a blood lactate analyzer (Lactate Scout+, EKF Diagnostics, UK). The area was cleaned first, using a dry tissue to remove sweat and then an alcohol swab. Once the area was dry, a lancet (often with a spring-loaded apparatus) was used to pierce the skin.

For CMJ height, as power is a critical component in many sports [25] and CMJ is a simple, practical, and reliable measure of power of the lower limbs, it would seem an obvious choice to measure and monitor performance. Additionally, CMJ has been directly linked with 0–30-m sprint performances [26] and relative strength during dynamic 1-RM squat and power clean [27], suggesting that those who perform better in the CMJ also perform better during sprint performances and 1-RM tests, such as the back squat and clean, are commonly used tests to assess soccer performance [28, 29]. The CMJ height was measured by using an iPhone app “My Jump” [30]. CMJ height was determined using an acknowledged flight-time calculation. During the CMJ, the subject rests their hands on their hips while performing a downward movement, followed by a maximum effort vertical jump. The subjects had to land in an upright position with knees bent on landing. Two trials were recorded and their average was used for analysis.

A 20-m single sprint is a standard test for assessing soccer players' running speed [28, 29, 31]. The test involved running a single maximum sprint over 20 m, with the time recorded using a stopwatch. Two trials were recorded and their average was used for analysis.

### 2.5. Statistical Analysis

Statistical analyses were performed using the SPSS software (version 21; SPSS Inc., Chicago, IL, USA). The analysis model was constructed to first rule out order effect and then analyze within-subjects effect of the intervention over all the time points. A 2 × 3 × 2 mixed model ANOVA was used, with two interventions and three time points within each intervention as within-subjects effects and crossover type as between-subjects effect. Mauchly's test was consulted and Greenhouse–Geisser correction was applied if sphericity was violated. Interaction term crossover type × intervention was observed to rule out order effect. Model residuals were tested for assumption of normality using the Shapiro–Wilk test. If within-subjects effect of intervention was significant, post hoc analysis with Bonferroni correction was used to observe differences between interventions in individual time points, after testing for normality. For within-intervention change of variable over time points, repeated measures ANOVA was used. The reliability of 20-m sprint was assessed by the intraclass correlation coefficient (ICC_3.1_). The descriptive data are presented as means ± standard deviations. A *P* value of <0.05 was taken as the significance level for all comparisons.

## 3. Results

Eighteen subjects were enrolled in this study, out of which four were excluded due to noncompliance. Therefore, fourteen subjects were recruited in this study to determine the acute effects of PLY and RES on PAP. The subjects were divided into two groups, one group (*n* = 7) performed PLY first and then RES, while the other group (*n* = 7) performed RES first and then PLY.

**Figure 1:**
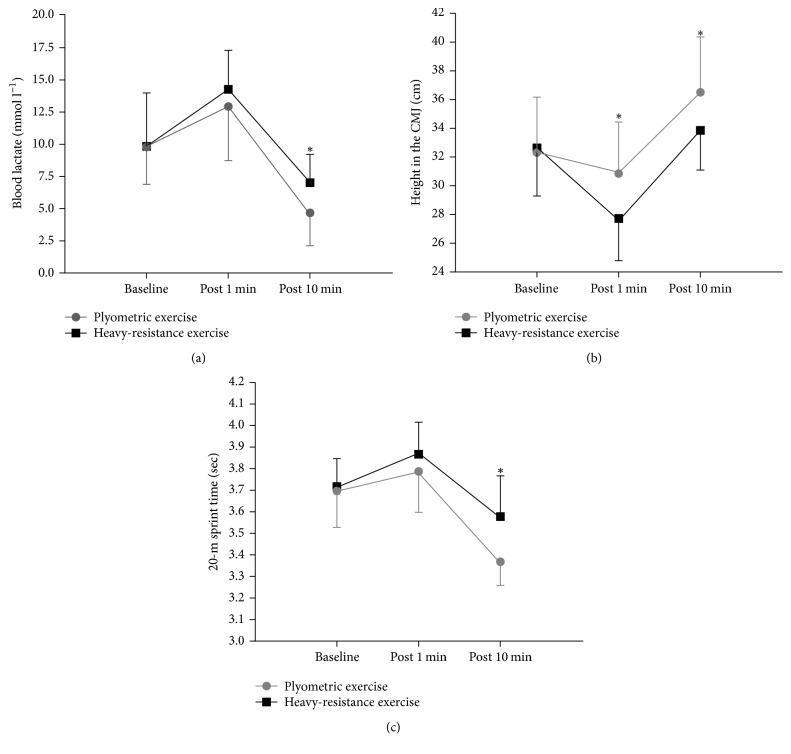
(a) Maximal blood lactate level for the PLY and RES group at the specified time intervals. Data are presented as mean ± SD. ^*∗*^Significant difference for *P* < 0.05. (b) Maximal height in the CMJ performance for the PLY and RES group at the specified time intervals. Data are presented as mean ± SD. ^*∗*^Significant difference for *P* < 0.05. (c) Maximal 20-m sprint time for the PLY and RES group at the specified time intervals. Data are presented as mean ± SD. ^*∗*^Significant difference for *P* < 0.05.

BLa was not influenced by protocol (time × protocol: *P* = 0.183) (Table 1). No significant differences were observed for BLa at baseline (*P* = 0.972) and after 1 min (*P* = 0.426). However, BLa were significantly lower for PLY after 10 min (*P* = 0.038) compared to those for RES (Table 2) (Figure 1(a)).

CMJ height was influenced by protocol (time × protocol: *P* < 0.001) (Table 1). At baseline, CMJ height was similar between protocols (*P* = 0.533). However, relative to baseline, CMJ height was significantly better for PLY after 1 min (*P* = 0.004) and after 10 min (*P* = 0.001) compared to that for RES (Table 2) (Figure 1(b)).

The 20-m single sprint test demonstrated high reliability (ICC_3.1_ = 0.93). The 20-m sprint time was influenced by protocol (time × protocol: *P* = 0.039) (Table 1). At baseline, no significant differences were observed for the 20-m sprint time (*P* = 0.487). There was no significant difference between PLY and RES after 1 min (*P* = 0.155). Furthermore, relative to baseline, the 20-m sprint time was significantly reduced for PLY after 10 min (*P* = 0.003) compared to that for RES (Table 2) (Figure 1(c)).

## 4. Discussion

A novel approach in this study was the measurement of the physiological response, that is, BLa, to establish the relationship between fatigue and potentiation. This study demonstrated that, in comparison to those at baseline, BLa were increased after 1 min and decreased after 10 min after both PLY and RES exercises, and there was a significant difference between the groups (PLY and RES) after a rest period of 10 min. In addition, this study compared the effects of PLY and RES in maximizing the jump and sprint performance in soccer players. The primary findings of this study indicate that an acute positive effect on CMJ height and 20-m sprint time was induced after both CAs. However, in comparison to that after RES, there was improved CMJ height after 1 min and after 10 min and reduced 20-m sprint time after 10 min after PLY.

The most striking result to emerge from the data was that, immediately after both exercises, when the BLa were higher, the CMJ and 20-m sprint performances were reduced; however, once the BLa were down, CMJ and 20-m sprint performances were remarkably improved. These results have further strengthened our confidence in the fact that fatigue and potentiation coexist, and if fatigue dominates, physical performance is decreased, but when potentiation dominates, physical performance is remarkably improved. Increase in muscle performance after a CA depends on the net balance between fatigue and potentiation, which can coexist at varying degrees after the completion of the CA [12]. Muscle performance may improve if potentiation dominates and fatigue is reduced, remain unchanged if fatigue and potentiation are at similar levels, or decrease if fatigue dominates.

This study demonstrated that, in comparison to that at baseline, BLa increased by 32.2% and 45% immediately after PLY and RES, respectively. However, after 10 min of recovery, it decreased by 52.2% and 28.6% after PLY and RES, respectively. The results of our study are consistent with those of previous studies in which BLa was measured after submaximal or maximal exercise [32–34]. Our results are also consistent with a previous study done by Street et al. [18], which reported that the lowest pH was seen 1 min after the exercise (5 min of one-legged knee-extensor exercise at power outputs of 30, 50, and 70 W), irrespective of the workload, after which interstitial pH recovered in a nearly exponential manner. Contrary to our expectations, we did not find a significant difference between the CAs. However, this is not particularly surprising given the fact that, apart from accumulation of lactate, ammonium and uric acid accumulation also contributes to development of fatigue.

Higher accumulation of lactate, ammonium, and uric acid, which are observed after an exercise protocol, results in a lower energy state of the muscle; the possible explanation for this could be that there is reduced or changed sensory feedback from the fatigued muscles after training and/or there could be increased activity of AMP deaminase enzyme, which hampers the increase in muscular free ADP concentration and thereby limits the contraction capacity of the muscle, which, in turn, pushes the muscle to higher levels of fatigue and, ultimately, higher energy disturbance [35]. Decreased ATP or PCr levels or both in the fast twitch or high-glycolytic fibers may also be a significant contributor to fatigue [36]. However, measurement of blood ammonia and uric acid levels and intramuscular ATP or PCr concentrations was beyond the scope of this study. But the relationships observed among BLa and physical performance allow us to state that when the BLa were high, fatigue dominated and the physical performance was decreased, but as the lactate started to subside, potentiation took over and led to an increase in physical performance above the baseline.

This study revealed that, in comparison to that at baseline, CMJ height decreased by 4.8% and 14.2% immediately after PLY and RES, respectively. However, after 10 min of recovery, it increased by 13% and 3.7% for PLY and RES, respectively. An increase in CMJ height after PLY has been observed in a previous study [20]; however, no significant difference after PLY was observed by Esformes et al. [14]. Similarly, RES has also increased CMJ height [37]. Greater improvement in CMJ height was observed after PLY, a possible explanation for this could be that PLY induces a lower level of fatigue compared to RES as it is associated with increased recruitment of type II motor units.

In contrast, a previous study [14] reported no significant difference between RES and PLY. The possible explanation for this could be that the volume of exercise used in these studies was too low to induce potentiation. Therefore, it was ineffective in the sufficient high recruitment of muscle fibers to improve the postsynaptic potentials. Moreover, Saez Saez de Villarreal et al. [11] reported an increase in potentiation after RES compared to that after PLY; the possible explanation for this could be that the volume of PLY was incapable of inducing similar levels of potentiation compared to RES. The results of our study prove that a volume of 40 jumps is an effective method to induce potentiation in CMJ performance.

Explosive muscle power is the main determinant of performance in many individual and team sports and can be successfully developed if the training consists of movements with high power output and maximum rate of tension development. CMJ performance is one of the most reliable and valid tests for the estimation of explosive power of the lower limbs [38]. As power is a critical component in many sports [25] and CMJ is a simple, practical, and reliable measure of power of the lower limbs, it would seem an obvious choice to measure and monitor performance. The CMJ height has also been directly associated with relative strength during dynamic 1-RM squat [27] and 0–30-m sprint performances [26]. A significant relationship was also observed between the team's average CMJ height and success [39]. These studies provide considerable insight into the importance of CMJ performance. The results from our study offer unprecedented evidence for incorporating PLY over RES to improve CMJ performance.

This study revealed that, in comparison to that at baseline, 20-m sprint time increased by 2.4% and 4% immediately after PLY and RES, respectively. However, after 10 min of recovery, it decreased by 8.9% and 3.7% after PLY and RES, respectively. This study demonstrated that PLY provides a more efficient method of inducing potentiation than RES. The results of our study are in agreement with those of previous studies which showed that sprint performance was improved after PLY [40, 41]. Sprint performance improvement after RES was also demonstrated by other previous studies [21, 42]. In contrast, a previous study [43] observed no significant improvement in sprint performance after the CA. However, the reason for this can be that the volume of exercise was too low to induce potentiation.

In accordance with the findings of the present study, plyometric conditioning seems to be a striking warm-up component but further investigations comparing its efficacy to traditional warm-ups are needed to consider adoption. Moreover, while plyometric preconditioning was associated with improved performance which leads us to speculate that PAP might be the operating mechanism, it is worth noting that PAP was not directly measured. Additionally, other physiological and psychological factors could play a role in determining voluntary performance. Therefore, future studies should utilise muscle twitch responses to assess potentiation and comment more conclusively on causation. Another important limitation of the study was the use of blood lactate as an indirect marker of fatigue. The study did not measure blood ammonia levels and intramuscular ATP or PCr concentrations as they require invasive procedure such as venepuncture and muscle biopsy. The physical performance tests (CMJ and 20-m sprint), although extensively used for assessment of soccer performance, do not truly reflect the repetitive, intermittent, high-intensity nature of the sport. Skill-based tests and multiple repeated sprints can be investigated to simulate actual game performance.

Our results demonstrated that the volume of exercise used in the present study might be effective in inducing potentiation. In a soccer game, high-intensity running is performed approximately every 70 seconds. The amount of high-speed running is what differentiates top-class players from those at a lower level. Therefore, increased sprint performance would contribute to better performance in the soccer game; however, explicit testing is required to observe better performance in a soccer game. The acute effects of plyometric and resistance CA were not seen during the competitive phase of the players to avoid the confounding effects of precompetitive anxiety and increased stress following competition but assessing actual match performance following preconditioning may provide a more realistic insight to its application in practical settings.

## 5. Conclusions

Plyometric CA seems to be a better option compared to heavy-resistance CA to achieve potentiation. Apart from the fact that RES induces less potentiation in comparison to PLY, the latter requires costly equipment (Smith frame) and RM calculation to make it individual-specific. In contrast, PLY seems to be less time consuming and highly efficient. Our study has led us to conclude that PLY is more efficient and effective for inducing potentiation compared to RES.

## Figures and Tables

**Table 1:** Summary of the results of two-way (2 × 3) repeated measures analyses of variance.

Dependent variable	Source	df	Partial *η*^2^	*F*	*P* value
BLa (mmoll^−1^)	Time	2	0.906	116.042	<0.001^*∗*^
	Protocol	1	0.086	1.123	0.310
	Time × protocol	2	0.132	1.827	0.183
CMJ (cm)	Time	2	0.940	187.899	<0.001^*∗*^
	Protocol	1	0.411	8.364	0.014^*∗*^
	Time × protocol	2	0.720	30.795	<0.001^*∗*^
20-m sprint (sec)	Time	2	0.922	141.679	<0.001^*∗*^
	Protocol	1	0.483	11.231	0.006^*∗*^
	Time × protocol	2	0.281	4.693	0.039^*∗*^

BLa: blood lactate; CMJ: countermovement jump; 20-m sprint: 20-metre sprint. ^*∗*^Significant at *P* < 0.05 level.

**Table 2:** Post hoc analysis.

Dependent variable	Time (min)	PLY	RES	Post hoc PLY versus RES (*P* value)
BLa (mmol l^−1^)	Baseline	9.79 ± 2.89	9.84 ± 4.16	0.972
	Post 1 min	12.94 ± 4.21	14.26 ± 3.04	0.426
	Post 10 min	4.68 ± 2.56	7.02 ± 2.19	0.038^*∗*^
CMJ (cm)	Baseline	32.34 ± 3.88	32.68 ± 3.38	0.533
	Post 1 min	30.78 ± 3.70	28.04 ± 3.24	0.004^*∗*^
	Post 10 min	36.55 ± 3.87	33.90 ± 2.77	0.001^*∗*^
20-m sprint (sec)	Baseline	3.70 ± 0.17	3.72 ± 0.13	0.487
	Post 1 min	3.79 ± 0.19	3.87 ± 0.15	0.155
	Post 10 min	3.37 ± 0.11	3.58 ± 0.19	0.003^*∗*^

PLY: plyometric exercise group; RES: heavy resistance exercise group; BLa: blood lactate level; CMJ: countermovement jump; 20-m sprint: 20-metre sprint. Data are presented as mean ± SD. ^*∗*^Significant at *P* < 0.05 level.
